# Evaluation of interprofessional teamwork modules implementation in an emergency department – A mixed-methods case study of implementation fidelity

**DOI:** 10.1186/s12913-021-06822-5

**Published:** 2021-08-21

**Authors:** Jenny Liu, Sari Ponzer, Nasim Farrokhnia, Italo Masiello

**Affiliations:** 1 Department of Clinical Science and Education, Södersjukhuset, Karolinska Institutet, Stockholm, Sweden; 2 Department of Computer Science and Media Technology, Linnaeus University, Växjö, Sweden

**Keywords:** Emergency department, Interprofessional, Teamwork, Implementation, Fidelity, Qualitative content analysis, Observations, Staff survey

## Abstract

**Background:**

The need for interprofessional collaboration has been emphasized by health organizations. This study was part of a mixed-methods evaluation of interprofessional teamwork modules implementation in an emergency department (ED), where a major intervention was didactic training of team roles and behaviours in combination with practice scenarios. The aim of the study was to evaluate the implementation of interprofessional teamwork modules from a staff perspective and focus on how implementation fidelity may be sustained.

**Methods:**

In this mixed-methods case study we triangulated staff data from structured observations, semi-structured interviews, and a questionnaire repeated at intervals over 5 years. A protocol of key team behaviours was used for the observations conducted in June 2016 and June 2018, 1½ and 3½ years after the initial implementation. A purposeful sample of central informants, including nursing and medical professionals and section managers, was interviewed from May to June 2018. The interview guide consisted of open-ended questions about the experiences of interprofessional teamwork modules and the implementation process. The questionnaire consisted of five statements about the perceived workload, interprofessional collaboration and patient satisfaction, where each was rated on a Likert scale.

**Results:**

Good fidelity to four out of five key team behaviours was observed during the first year. However, fidelity was sustained only for one key team behaviour after 3 years. We conducted a qualitative content analysis of 18 individual interviews. The theme *Enjoying working together, but feeling less efficient* emerged of the interprofessional teamwork modules, despite shorter ED stays for the patients. Negative experiences of the staff included passive team leaders and slow care teams. The theme *Stimulating to create, but challenging to sustain* emerged of the implementation process, where barriers were not adressed and implementation fidelity not sustained. The staff questionnaire showed that the perceived work conditions was improved in periods of high fidelity, but deteriorated to pre-implementation levels as fidelity to the key team behaviours decayed in 2018.

**Conclusions:**

Extensive planning and successful initial implementation were not enough to sustain the key behaviour changes in the study. The use of implementation frameworks can be helpful in future projects.

## Background

In 1972, leaders of major health professions agreed on the need to train and educate health professionals for collaborative practice [1]. This has been repeatedly been emphasized over the past five decades by the Institute of Medicine and the World Health Organization [2–5]. Collaborative practice of health professionals is needed since medical errors are correlated to failures of teamwork and communication, while effective teamwork improves outcomes in healthcare [5]. Studies of interprofessional teamwork including teamwork training in emergency department (ED) settings have reported reduced error rates, reduced waiting times to physician assessment, shorter ED lengths of stay, and improved patient and staff satisfaction [6–12].

However, these studies have evaluated teamwork interventions using limited time frames ranging from 3 to 18 months, as per similar studies in other healthcare settings. This contributes to a knowledge gap of how effective healthcare teams may be sustained [13]. Therefore, the authors of Cochrane reviews recommend the use of longer follow-up periods and mixed methods to examine interprofessional collaboration interventions [14, 15]. Moreover, the outcome of an intervention depends on whether it is implemented with fidelity over time, i.e., complying with the standards that were developed for the intervention [16, 17]. Implementation research seeks to bridge the gap from research evidence to health practice and frameworks have been developed to increase the success rate of an implementation with sustained fidelity [18]. Some examples are the Quality Improvement Framework [19], the Theoretical Domains Framework of behaviour change [20], and the Active Implementation Frameworks [21].

This study is part of a mixed-methods evaluation of the implementation of interprofessional teamwork in a large ED, which was reorganized into teamwork modules with integrated triage of patients instead of a separate triage section. We have previously reported improved patient waiting time and ED length of stay during the first year after the implementation of interprofessional teamwork modules [11, 12], while in this study we evaluated the implementation process over a period of nearly 5 years by using data from the health professionals. The aim was to examine the degree of fidelity to the teamwork implementation and explore the experiences of health professionals to answer our research questions: Was the implementation fidelity sustained? How did the health professionals perceive the interprofessional teamwork modules?

## Methods

### Study design, setting and participants

We conducted a mixed-methods case study from October 2013 to June 2018 of the interprofessional teamwork modules implementation at the adult ED of Södersjukhuset, a 600-bed urban teaching hospital in Stockholm and the busiest ED in Sweden with 110,000 patient arrivals annually. Emergency medicine is a young specialty in Sweden, which means that most EDs depend on junior physicians on rotation from other departments.

However, the study ED was first in Sweden to introduce a residency program for emergency medicine, so that ten emergency specialists and 50 junior physicians belonging to the ED covered 60% of the shifts by the start of the study in 2013. The remaining shifts still depended on physicians from the internal medicine and cardiology departments, who only treated patients with main complaints related to their respective disciplines. Adding the interns and primary care residents, a total of 500 individual physicians worked in the ED each year. All nursing staff, 120 registered nurses and 60 nursing assistants, belonged to the ED. In summary, the study population consisted of 180 nursing and 60 medical staff belonging to the ED, and more than 400 additional medical staff on rotation from other departments.

Before the introduction of teamwork modules, patients were first seen at the triage section by registered nurses, who prioritized them according to the Rapid Emergency Triage and Treatment System (RETTS) [22] and then directed them to one of three desks, internal medicine, cardiology or emergency medicine, depending on the main complaint. There, the next available doctor assessed the patient and left written orders for the next available nurse to carry out. The working hours differed between the health professions and between the departments for physicians, so that each nurse carried out the orders of several doctors and each doctor communicated with several nurses during a shift. Due to an increasing ED length of stay [11], the hospital management decided in October 2013 to improve patient flow and work conditions at the ED by introducing interprofessional teamwork modules.

### The innovation – interprofessional teamwork modules

**Fig. 1:**
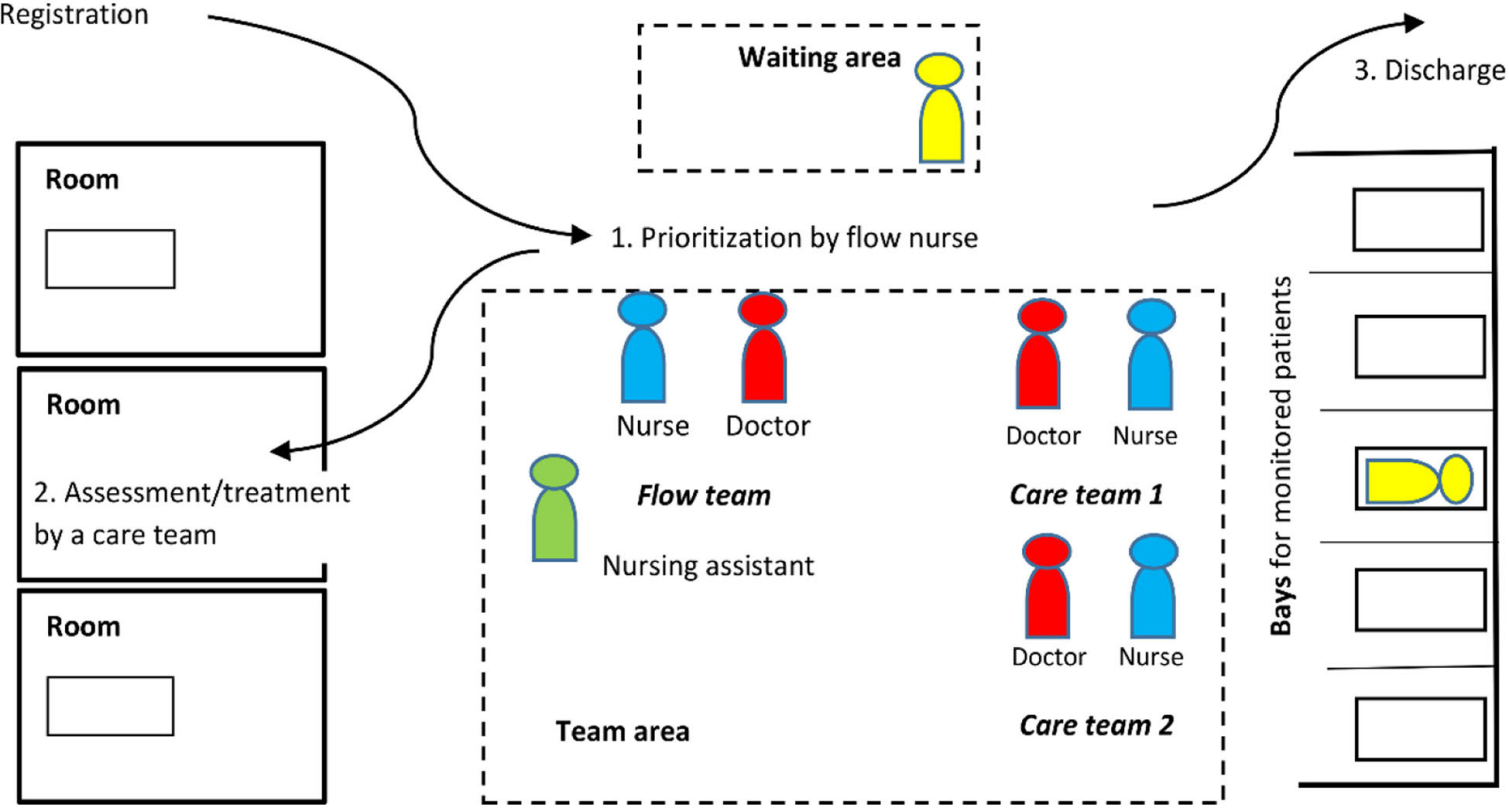
Interprofessional teamwork in a module. Each module had dedicated rooms, bays, a waiting area, and a team area. A module was staffed by a leading flow team and two care teams. Each team consisted of a nurse and a doctor, with the most senior pair forming the flow team. A nursing assistant helped all team members. Reproduced with CC BY-NC 4.0 license

Each teamwork module had dedicated rooms, bays, a waiting area, and a team area. A module was staffed by a leading flow team and two care teams. Each team consisted of a registered nurse and a physician, with the most senior pair forming the flow team. After registration, the patient was directed to a module, where the flow team nurse prioritized and was responsible for the queueing patients until a care team started to assess the patient. After taking the patient history and carrying out the physical examination, the care team decided on a plan together with the patient and the flow team doctor. The care team then carried out diagnostic and treatment activities in an immediate sequence, before leaving the room ready for the next patient (Fig. 1).

### The implementation process

**Fig. 2:**
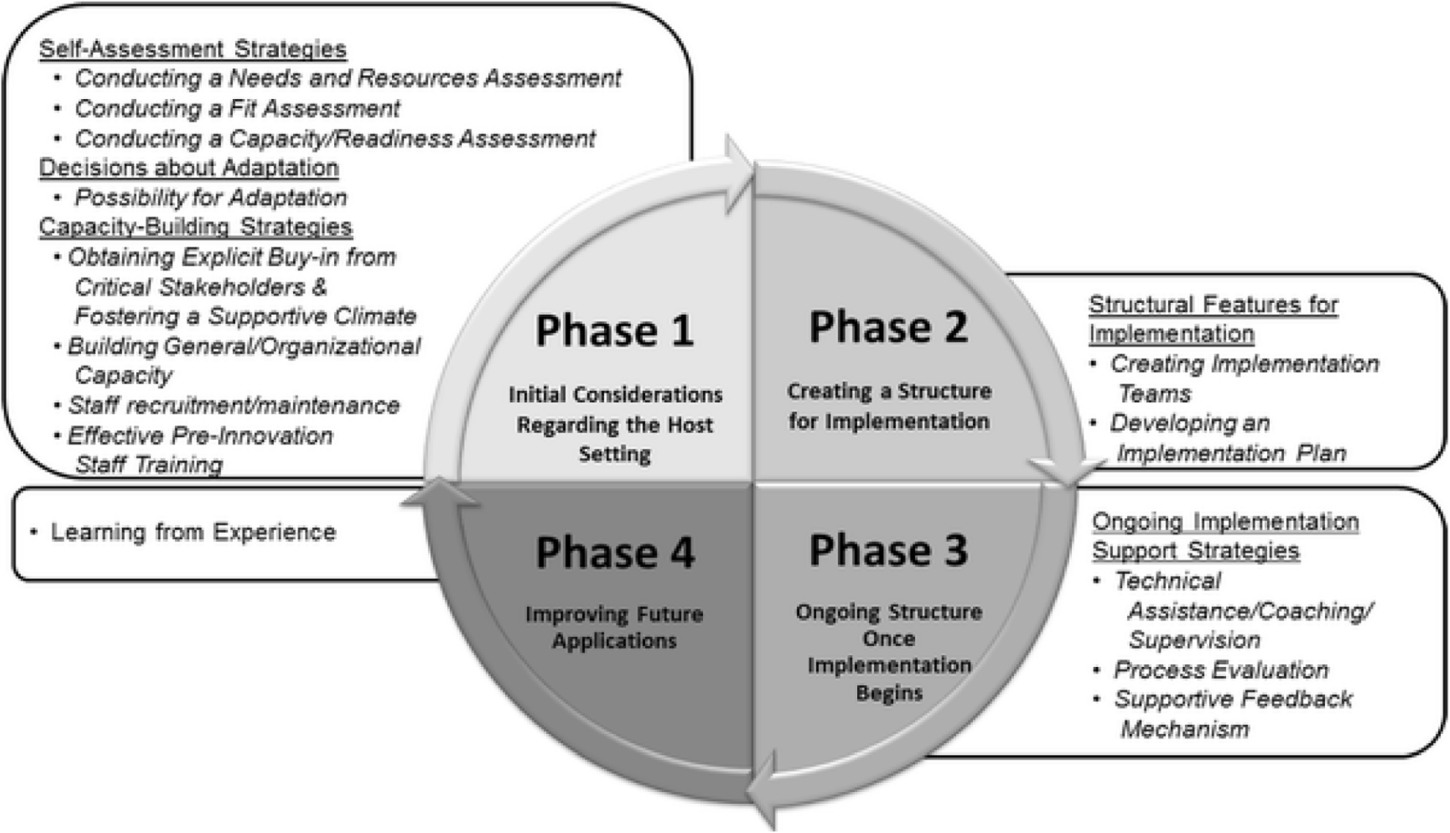
The Quality Implementation Framework by Meyers et al. Reproduced with permission

In 2013, we were not aware of the Quality Implementation Framework (QIF) published the previous year by Meyers et al. [19] (Fig. 2), which would have been useful when planning and conducting the implementation of interprofessional teamwork. Instead, we describe the process according to the steps in the four phases of QIF.

#### Phase 1 – initial consideration regarding the host setting

##### Build capacity

The hospital chief executive officer recruited an external facilitator with expertise from teamwork implementation in other hospitals. Five department managers each assigned one senior physician 20% of full time to be ambassadors at their respective departments and lead the three multi-professional improvement groups, focusing on cardiology, internal medicine, and surgery, respectively. In total, 60 group members consisting of physicians, registered nurses, nursing assistants and section managers participated in these groups.

##### Self-assess

The improvement groups identified a need for improved collaboration between the medical and nursing staff, and for improved medical support on the spot. All management levels found a good fit of interprofessional teamwork, hereafter referred to as the innovation, to the needs identified. However, the innovation would require more specialists or senior residents from four departments other than the ED.

##### Decide adaptation

The improvement groups conducted ten Plan-Do-Study-Act-cycles [23] of the innovation from November 2013 before its introduction on weekdays in November 2014. Additional test cycles were conducted before the introduction for night shifts in November 2015. Since there were less physicians on weekends, additional physician shifts were required to form teams with the nursing staff when the innovation was introduced in February 2016. The additional cost was financed by reducing and modifying the generic team staffing and the modifications differed depending on medical specialty and day of the week. Additional modifications followed during the summer vacations in 2016, when nursing assistants replaced registered nurses in many care teams. The replacement was maintained for several care teams after the summer period, due to a continuous shortage of registered nurses. By October 2016, all these staff modifications had increased the number of team varieties from 2 to 13.

##### Build capacity

The ED facility, including the triage section, was reorganized into teamwork modules (Fig. 1). Doctors moved from back offices to the shared team area, where each member had a dedicated workplace. Work schedules were synchronized between the professions, enabling team members to start and end a shift together [11]. In September 2014, all ED staff participated in a pre-implementation training, consisting of a one-hour lecture followed by practice scenarios. The roles and responsibilities of each team member were explained in the lecture and then the health professionals played their own team roles in the scenarios, where some staff members played the role of patients. An instructor facilitated the simulations, starting with routine cases and then added challenging scenarios suggested by the participants. Challenging scenarios could be a patient suffering from domestic violence or a junior physician disagreeing with the senior physician about the care plan. In addition to the training of the ED staff, 200 residents and specialists from other departments participated in two-hour lunch sessions in the fall of 2014, where they were joined by ED nurses after the lecture to practice scenarios.

#### Phase 2 – creating a structure for implementation

##### Create implementation teams

The physician ambassadors, nursing unit leaders, and section managers formed implementation teams.

##### Develop plan

The innovation was introduced on weekdays from 8 am to 9 pm as a first step in November 2014. The night shifts followed in November 2015, and the weekends in February 2016.

#### Phase 3 – ongoing structure once implementation begins

##### Ongoing support

**Fig. 3:**
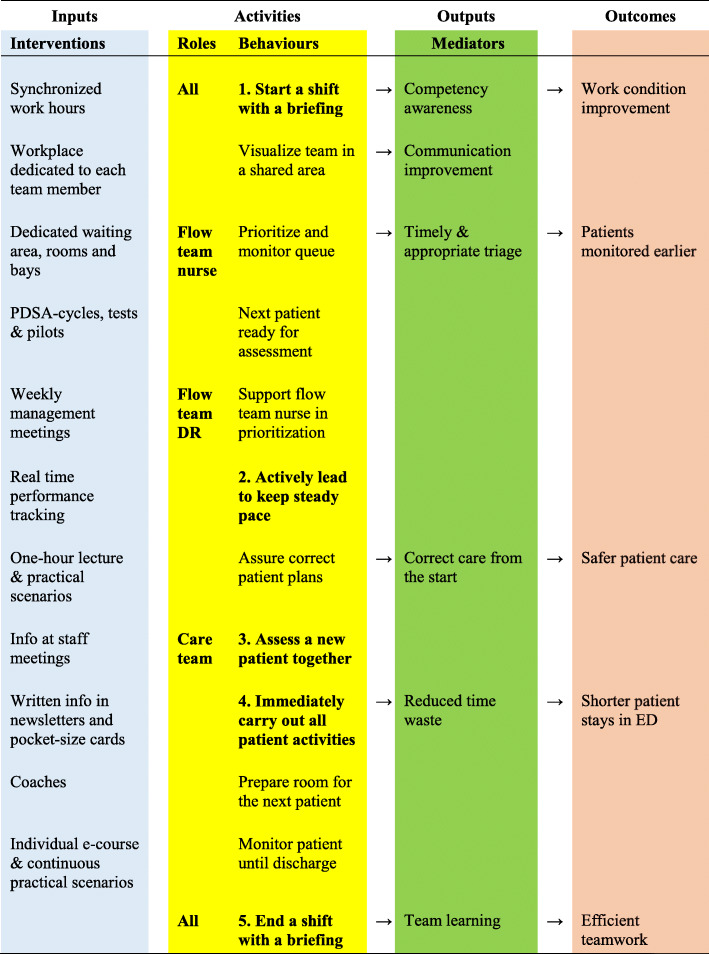
Logic model of the interprofessional teamwork implementation. Of the team behaviours identified in multiprofessional workshops, those numbered 1 to 5 were the core components of the teamwork innovation

The major implementation intervention was a team training package like the pre-implementation training delivered in the fall of 2014. The hospital Clinical Training Centre hosted two workshops in March 2015, where representatives of the health professions achieved consensus on the essential behaviours for each team role and the core components of the innovation (Fig. 3).

A one-week instructor course was organized in April 2016 and a 60-min individual e-course was launched in January 2017, which all staff members were requested to complete. From January 2017, team members of a module were invited to a practical training session before starting their evening shift together. However, these training sessions turned into discussion groups due to poor physician attendance. Therefore, lunch meetings were arranged in the spring to motivate the physicians to attend the practical training sessions. However, the training sessions were not resumed after the summer break as the department manager and several section managers resigned in the fall of 2017.

##### Supportive feedback mechanism

Another implementation intervention was the development of the local electronic ED tracking system allowing teams to track their performance in real time. It also allowed the implementation team to access and present performance data in monthly newsletters. However, this functionality was lost in April 2016 when a system generic to all hospitals in the Stockholm County replaced the local tracking system. Moreover, coaches could only be scheduled for 1 week at the introduction on weekdays and for a few shifts on nights and weekends, due to manpower limitations.

##### Evaluate process

Hospital administrators presented data of patient throughput, such as the waiting time for physician assessment and ED length of stay, at weekly meetings for review by managers at all level. A 5-item staff questionnaire of the perceived work condition was distributed before the introduction, at each introduction step, and in June 2018. Structured observations of team behaviours were conducted in June 2016 and repeated in June 2018. Semi-structured interviews of the staff and section managers were carried out from May to June 2018.

#### Phase 4 – improving future applications

##### Learn from experience

We describe this final step in the results and discussion sections.

We visualize the implementation strategy used to enhance team behaviours identified as the core components of the innovation, and the expected mechanisms to improve patient outcomes in a logic model. (Fig. 3).

### Data collection and analysis

#### Structured observations

**Fig. 4:**
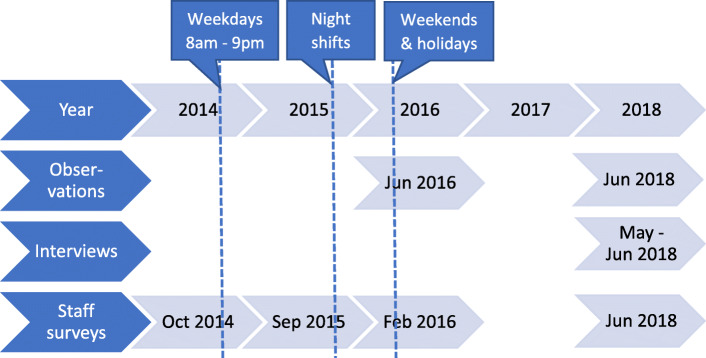
Timeline of the data collection

In June 2016 and June 2018, the lead author conducted observations where the team members performed their tasks without being disturbed (Fig. 4). The observed teams were chosen to cover all specialties at the ED, which were cardiology, internal medicine, orthopedics, and general surgery. The observed teams were not notified in advance, but observations started only after individual consents by all team members. The observation protocol consisted of the team behaviours that were identified as learning objectives of the team training package during the multiprofessional workshops. The observed behaviours were either dichotomic or assessed on a Likert scale from 1 (strongly disagree) to 5 (strongly agree). Five of these behaviours were considered the core components of the teamwork innovation: 1. All team members in a module starting a shift with a briefing; 2. The flow team doctor actively leading the module; 3. Doctor and nurse in the care team joining for the first assessment of a patient; 4. The care team immediately carrying out all patient activities once the plan was decided; 5. All team members in a module ending a shift by a briefing.

#### Semi-structured interviews

A purposeful sample of central informants who had experienced the implementation process was invited to participate in semi-structured interviews. The informants were chosen to represent all health professions and team roles, including physicians from five departments and the ED nursing staff. Section managers and members of the improvement groups of both genders were also represented. The interviews were conducted individually from May to June 2018 (Fig. 4). The interview guide, also provided as Additional file 1, consisted of four open-ended questions about the experiences: 1. Of the new teamwork process; 2. From the planning period; 3. From the initial implementation period; 4. From the period that followed. If needed, the researcher asked about what worked well or did not work, and what would make it better. The voice recordings, ranging from 30 to 60 min in length, were transcribed verbatim and then read and discussed by all authors using an interpretative epistemological approach. We conducted a qualitative content analysis according to Graneheim and Lundman [24, 25].

All observations and interviews were conducted by the lead author, who had been a clinician and section manager at the ED until June 2013, prior to the study. She became one of the improvement group leaders from the start in October 2013, but handed over the position in April 2016 and only performed research activities from May 2016. The lead author performed the condensation and coding of meaning units, then all authors reached consensus of the final subcategories, categories and themes of the content analysis.

#### Staff questionnaire

Staff surveys were conducted from 27 to 31 October 2014 before the innovation was introduced, after the introduction for weekdays from 7 to 11 September 2015, for weekends on 6, 7, 13 and 14 February 2016, and 3½ years after from 11 to 17 June 2018 (Fig. 4). Staff members with work shifts during the survey periods were included and those working multiple shifts were encouraged to fill out a new questionnaire form at the end of each shift. The implementation team reminded the staff and collected the questionnaire forms by the end of each work shift, except for June 2018 when the team was no longer active.

The same questionnaire, also provided as Additional file 2, was distributed on paper and consisted of five statements about the work shift: 1. *I had an acceptable workload*; 2. *I had enough time to carry out my tasks*; 3. *I experienced that nursing assistants, registered nurses and doctors collaborated well*; 4. *I experienced that my patients were satisfied with the care they received*; 5. *In summary, I had a good work shift today*. Each statement was rated on a Likert scale from 0 (strongly disagree) to 5 (strongly agree). The questionnaire also offered a blank space for free comments. Data from each questionnaire were transferred to Microsoft® Excel and IBM® SPSS® Statistics version 26. We used the Chi-squared test for the proportions of negative (0, 1 or 2), neutral (3), and positive (4 or 5) ratings to analyse differences between the periods. We calculated the response rate from the total number of scheduled work shifts for the medical and nursing staff during each survey period.

## Results

### Structured observations

In June 2016, team behaviours were observed for 50.5 h during seven sessions, including two in the evening and one on a holiday. This means that seven different teamwork modules were observed during the entire shift, and 12 additional teamwork modules only for 1½ - 6½ hours during the overlap time and handover. In June 2018, the observations were stopped after 37.5 h, when five teamwork modules observed during the entire shift and four additional teamwork modules during the overlap time all had a similar team performance.

In 2016, all 17 (100%) teams observed during the start of a shift held a briefing, whereas only one out of nine (11%) teams observed at the end of a shift gathered for a briefing. In 2018, eight out of nine (89%) teams observed during a shift start held a briefing, whereas none of the five teams observed at the end of a shift gathered for a debriefing. The scores of the remaining three team behaviours decreased in a more significant way. In 2016, 7 out of 14 (50%) teams scored four or five for *Active leader*, 5 out of 14 (36%) teams for *Joint first assessment*, and 10 out of 17 (59%) teams for *Immediate patient activities*. In 2018, no team scored four or five for any of these behaviours (Table 1).

**Table 1:** Structured observations of team behaviours in Jun 2016 and Jun 2018

	Jun 2016						Jun 2018					
Observation hours/sessions (N)	50.5/7						37.5/5					
Complete + partial teams (N)	7 + 12						5 + 4					
**Dichotomic**	**Yes**	**No**				**NA**	**Yes**	**No**				**NA**
Start with a briefing (N)	17	0				2	8	1				0
End with a briefing (N)	1	8				10	0	5				4
**Scores**	**1**	**2**	**3**	**4**	**5**	**NA**	**1**	**2**	**3**	**4**	**5**	**NA**
Active leader (N)	1	2	4	2	5	5	2	0	3	0	0	4
Joint first assessment (N)	4	1	4	1	4	5	5	0	0	0	0	4
Immediate patient activities (N)	0	4	3	4	6	2	7	1	0	0	0	1

Two variables were dichotomic and the other three were each assessed on a Likert scale from 1 (strongly disagree) to 5 (strongly agree)

*Abbreviations*: *N* number, *NA* not assessed

### Semi-structured interviews

We invited 26 informants and 21 (81%) accepted to participate, while the remaining five did not respond. In the end, we included 18 interviews for qualitative content analysis, since two informants could not find time for the interview and one declined voice recording during the interview. The analysis included interviews of ten female and eight male informants, who were registered nurses (*N* = 10), physicians (*N* = 7) and nursing assistant (*N* = 1). Among these, two physicians and three registered nurses were section managers.

We classified the content into two separate areas, the teamwork and the implementation. The teamwork area consisted of two main categories, positive and negative experiences of the new work process, including those relating to the staff fidelity to the interprofessional teamwork modules. The main categories of the implementation area were experienced enablers and barriers, including those relating to the organisational fidelity to the implementation strategy. Each main category consisted of several subcategories (Table 2).

**Table 2:** Qualitative content analysis of 18 individual interviews

**Teamwork area**	**Positive experiences**	**Negative experiences**
	Work together	Time consuming
	Communicate easier	Noisier workplace
	Distribute queue	Queue awareness
	Right from start	Demanding for seniors
Staff fidelity	Important briefing	Passive flow team doctors
	Share team area	Slow care teams
		Flow team nurse overload
		Pointless debriefings
*Theme*	*Enjoying working together*,	*but feeling less efficient.*
**Implementation area**	**Enablers**	**Barriers**
	Top manager support	Physician involvement
	Enjoy improvement groups	Around the clock
	External facilitator	Coaching
	Statistics supply	Five departments
		Novice nurses
Context fidelity		Competency mix not met
		Technical support withdrawn
		Team training discontinued
		Budget adaptations
		Increasing in-bed occupancy
*Theme*	*Stimulating to create*,	*but challenging to sustain.*

The content was classified in two areas, teamwork and implementation. Main categories of the teamwork area were positive and negative experiences of the new work process, including those relating to the staff fidelity. Main categories of the implementation process were enablers and barriers experienced, including those relating to the context fidelity. Each main category consisted of several subcategories. One theme emerged from each content area

### The teamwork area

All informants appreciated working together in teams. A majority mentioned improved communication and quality of care, such as deciding correct plans from the start. Although all professions mentioned a calmer atmosphere, the flow team doctor role was demanding and required leadership skills. Moreover, moving from the back offices to share a team area with the nurses meant noisier workplaces for physicians and a new stressful awareness of the queueing patients. Abandoning the comprehensive triage process and spreading the responsibility of queueing patients to flow team nurses in several modules improved the patient safety. No nurses wanted to go back to the old triage process, while physicians experienced a loss of control without the information previously collected during the triage process.

#### Staff fidelity to the interprofessional teamwork modules

The team briefing at the beginning of a work shift was important in order to know what to expect from each other. The dedicated workplace was appreciated, despite the location in a cramped and noisier team area. Physicians no longer had to find an available workplace or a colleague willing to take over patients. Nurses felt that doctors shared the responsibility when they also were located in front of the patients. However, some could not see the point of a briefing at the end of a shift, while others found it difficult to interrupt their tasks for it.

All professions stressed the poor fidelity to an active leading role by the flow team doctors, due to the fact that specialists from other departments were unwilling to work in the ED, and the existing culture to let junior physicians assess patients on their own and consult senior physicians only when they needed. In addition, the coordination required to join each other for the first assessment of a patient was difficult and all professions experienced waiting for each other. During the patient history and physical examination, the care team nurse tended to other patients instead, especially after being assigned queueing patients in the spring of 2018. While nurses were frustrated over slow junior doctors, physicians complained of inexperienced nurses and asked for more nurses. Nurses considered that the ED was ready for flexible care teams consisting of two doctors and two nurses, while physicians wished to keep their individually dedicated nurses.

The theme *Enjoying working together, but feeling less efficient* emerged from the teamwork area. We exemplify with a few quotes:

*Many (of my colleagues) said it was fun working together with a patient. Together in a team.*

*–* Physician, male (Informant 2)*.*

*We have learned to know each other and it makes the work a lot easier.*

*–* Registered nurse, male (Informant 11)*.*

*On the other hand, we are supposed to coordinate and go to the patient, sometimes taking the flow team doctor along if we have an intern. And that takes more time.*

*–* Physician, female (Informant 14).

*Feels like we are producing much less in this system than before.*

*–* Registered nurse, female (Informant 17).

### The implementation area

During the planning phase, members of the improvement groups felt that the hospital was focused on the ED. They enjoyed planning across departmental and professional borders. Section managers felt they were given a clear direction from the hospital chief executive officer. However, physicians did not feel involved and some felt that managers handpicked members to the improvement groups. Physicians experienced missing or incorrect information but also too much information when the innovation was launched, while the nursing staff felt well informed. Section managers stressed the difficulties to reach out to everyone, especially to the night shift staff. The transition from the new teamwork process of the evening shift to the old work process of the night shift was described as chaotic and unbearable, which could have been avoided by a one-step introduction. The long-awaited final introduction for weekends required modifications of the module staffing and the resulting varieties were confusing. Coaches should have been scheduled for much longer periods. Moreover, nurse coaches were not comfortable to coach doctors, nor were physicians comfortable to coach colleagues from another department.

Many informants discussed a simultaneous project recruiting registered nurses fresh from their examinations to the ED. Previously, 2 years’ work experience were required. Physicians mistrusted inexperienced nurses who failed to prepare patients or execute orders. The junior nurse gladly acquired knowledge from the care team doctor but missed the opportunity of having senior nurses as professional role models. As a consequence, the managers noticed a reluctance among these nurses to be promoted as a flow team nurse. Section managers wished that the three improvement groups had been fused earlier to prevent the diverse module varieties created by the separate groups. However, activities in the improvement groups faded out after the final introduction for weekends in February 2016.

#### Context fidelity to the implementation

Junior residents were scheduled as flow team doctors, due to a lack of specialists and senior residents. In addition, the competency mix of other team roles was not maintained, so that a junior nurse had the senior flow team nurse role or both care teams were staffed by physicians during internship instead of a resident in one of the care teams. Some staff members missed the real-time performance tracking system and the external facilitator after both were withdrawn in April 2016. Nurses wished that managers had been more active when physicians failed to attend the practical training sessions. Care teams stopped using the computers in the patient rooms due to a slower log-in procedure of a new security system. The number of flow teams was reduced in a budget cut in the spring of 2018, and the remaining flow team nurses assigned queueing patients to care teams. An increasing in-bed occupancy rate due to a lack of ward nurses meant that care team nurses were busy tending to the boarding patients.

The theme of the implementation area was *Stimulating to create, but challenging to sustain,* which can be exemplified by the following quotations:

*It was very stimulating and fun to think new about …*. *Working across the formerly so strict department borders was fun and one felt hopeful being able to do something …*

– Physician, male (Informant 3).

*We don’t get specialists any more, hardly any residents. So the competence level has been lower, making it boring for nurses who think it works too slowly.*

– Registered nurse, female (Informant 13).

### Staff questionnaire

The overall response rate was 46, 52, and 39%, respectively, for the first three survey periods and was similar for the medical and nursing staff. Without reminders at the end of work shifts in the 2018 survey period, the overall response rate was only 7% (Table 3). Before the teamwork introduction, only 49 to 52% agreed or strongly agreed with the three statements *Acceptable workload, Enough time,* and *Good work shift*. After the introduction for weekdays, over 85% agreed or strongly agreed with all five statements in the 2015 survey period. Despite a high patient inflow, 32 out of 89 free comments mentioned calm work shifts with low numbers of patients. The ratings decreased slightly after the introduction on weekends in 2016. By June 2018, the ratings had decreased to pre-implementation levels (Table 3)*.* Eight out of ten free comments wrote about chaos, patients annoyed over long waiting times, and inefficiency.

**Table 3:** Staff questionnaire of the perceived work condition from 2014 to 2018

		2014	*p*-value	2015	*p-*value	2016	*p-*value	2018	*p-*value
		Before		Weekdays		Weekends		After 3.5 years	
Responses (N)		299		352		156		54	
Overall rate (%)		46		52		39		7	
Medical/Nursing (%)		44/41		51/51		35/40		2/10	
***Item***	***Rating***								
**Acceptable workload**	**Negative**	30%	< 0.01	5%	< 0.01	12%	< 0.01	43%	0.31
	**Neutral**	20%		8%		17%		15%	
	**Positive**	49%		87%		71%		43%	
	**Missing(N)**	1		2		1		0	
	**Mean**	3.2		4.5		4.0		2.8	
**Enough time**	**Negative**	28%	< 0.01	5%	< 0.01	13%	< 0.01	33%	0.78
	**Neutral**	21%		7%		17%		22%	
	**Positive**	51%		88%		69%		44%	
	**Missing(N)**	1		3		1		0	
	**Mean**	3.3		4.5		4,0		3.0	
**Good collaboration**	**Negative**	11%	< 0.01	3%	0.55	4%	0.10	4%	0.20
	**Neutral**	15%		6%		4%		13%	
	**Positive**	72%		91%		92%		83%	
	**Missing(N)**	7		2		0		0	
	**Mean**	4.0		4.6		4.6		4.2	
**Satisfied patients**	**Negative**	19%	< 0.01	3%	0.21	6%	< 0.01	22%	0.40
	**Neutral**	17%		8%		11%		24%	
	**Positive**	64%		88%		81%		54%	
	**Missing(N)**	3		3		3		0	
	**Mean**	3.6		4.4		4.3		3.5	
**Good work shift**	**Negative**	24%	< 0.01	5%	< 0.01	10%	< 0.01	33%	0.40
	**Neutral**	23%		8%		17%		17%	
	**Positive**	52%		86%		73%		50%	
	**Missing(N)**	3		3		0		0	
	**Mean**	3.4		4.4		4.0		3.2	

A 5-item questionnaire was distributed on paper at intervals. Each item was rated on a Likert scale from 0 (strongly disagree) to 5 (strongly agree). Staff members with work shifts during the survey periods were included and those working multiple shifts were encouraged to fill out a new form for each shift. The overall response rate and per profession were calculated from the total number of work shifts for each period. Differences between the periods were analysed by the Chi2-test of the proportions of negative (0, 1 or 2), neutral (3), and positive (4 or 5) ratings

## Discussion

This study evaluated the implementation of interprofessional teamwork modules in an ED, where the major intervention was team training using a didactic training in combination with practice scenarios. The study focused on fidelity to the core components of the teamwork innovation. From the structured observations we found a good staff fidelity to four out of five key team behaviours 1½ years after the introduction, but staff fidelity remained only for one behaviour after another 2 years. The semi-structured interviews exposed negative expriences of the staff, including barriers that were not fully addressed by the organisation to sustain the fidelity. From the staff questionnaire, we found that the perceived work load was low when the observed staff fidelity was high, and returned to pre-implementation levels when it had decayed in 2018.

Mazzocato et al. [26] observed team behaviours during the second week after the implementation of interprofessional teamwork in another Swedish ED. The planned behaviour in that study, taking patient history with all team members present, was observed for only 36% of the patients in the internal medicine section. In the surgery section, team behaviours were hardly observable due to a different implementation approach [27]. In comparison, the present study found a higher initial staff fidelity to the corresponding behaviour, *Joint first assessment*.

On the other hand, Ajeigbe et al. [6] conducted a cross-sectional staff survey comparing four EDs that implemented teamwork structures after the Emergency Team Coordination Course (ETCC) in the MedTeams™ project to four control EDs without the ETCC training and teamwork structures. Ten years after the ETCC, the authors found that the perceived job environment, autonomy, and control over practice was better in the intervention EDs compared to the control EDs. In the present study, we also found a better perceived work condition after 1 year, but most of the improvement was lost after another 2 years. However, a strength of the present study is the triangulation of data from longitudinal observations and in-depth interviews.

Sustained behaviour change is essential in implementation practice [28, 29]. Use of the Theoretical Domains Framework of behaviour change, developed by collaborating behaviour scientists and implementation researchers [20], may have prevented the decay of key team behaviours we observed after additional 24 months. For example, senior physicians could have been engaged to explore what they needed in terms of capability, opportunity and motivation to change behaviour (COM-B) from being a passive consultant to an active leader. This COM-B diagnosis would have identified barriers to actively lead a teamwork module, such as unwillingness to work in the ED and their attitudes towards junior physicians. If such barriers were adressed appropriately, then the poor leadership of the flow team doctors that we found in the observations and the interviews would have been preventable.

The identified barriers could also be linked to the Active Implementation Frameworks [21] and its competency drivers be used to choose effective interventions. For instance, by selecting motivated senior physicians with leadership skills or training and coaching them in such skills. To effectively deliver these compentency drivers, the organisation drivers selecting qualified staff to be trained as coaches and scheduling the training would have been helpful. The lack of qualified staff and economic resources could then be identified earlier and adressed differently, for example, by a gradual up-scaling of the number of teamwork modules. Moreover, the withdrawal of the real-time performance tracking system, computer log-in problems, and the increasing hospital occupancy rate could have been adressed more actively by using other organisation drivers, such as the use of decision support data system at every level and interventions to overcome system barriers. Finally, use of the leadership drivers could help to transform the culture and carry on the team training.

The study shared some limitations with other qualitative studies. We sought to limit the risk of observer bias by using a pre-defined protocol and the same observer in both periods. We considered that the risk of power imbalance between the observer/interviewer and the participants was low, since 3 to 5 years had elapsed after the lead author resigned as a section manager. We adressed the risk of interpretation bias by all authors reading and discussing the interview transcripts, and by data triangulation. A home-made staff questionnaire was chosen, because it was brief and simple enough to allow repeated measurements in a busy ED. Finally, the findings from this single-site study may not be fully transferable or generalisable to different ED settings.

## Conclusions

Despite a successful initial implementation with improvements of perceived work condition in this study and improved patient throughput in our previous studies, four out of five core components of the interprofessional teamwork modules innovation were hardly observable 3½ years after the introduction. Qualitative content analysis of the interviews exposed implementation fidelity issues that could be managed in future projects by a systematic use of implementation frameworks.

## Data Availability

The datasets are available from the corresponding author on request.

## Abbreviations

COM-B: Capability, opportunity and motivation to change behaviour
ED: Emergency department
ETCC: Emergency Team Coordination Course
N: Number
